# From candidate genes to omics: Unbiased approaches reshaping arthropod Evo-Devo

**DOI:** 10.1590/1678-4685-GMB-2025-0217

**Published:** 2026-06-26

**Authors:** João Vieira, Rodrigo Nunes-da-Fonseca

**Affiliations:** 1 Universidade Federal do Rio de Janeiro, Instituto de Biodiversidade e Sustentabilidade, Macaé, RJ, Brazil.; 2 Institute of Zoology, Köln, Germany

**Keywords:** Arthropoda, axis patterning, chromatin accessibility, evolutionary developmental biology, single-cell, Transcriptomics

## Abstract

*Drosophila melanogaster* established the candidate-gene paradigm that shaped arthropod evolutionary developmental biology (Evo-Devo) for decades. Genome-wide methods-bulk RNA-seq, single-cell/single-nucleus transcriptomics, chromatin profiling (ATAC-seq, CUT&Tag/CUT&RUN), and 3D genome mapping (Hi-C)-now enable direct interrogation of gene regulatory networks (GRNs) in non-model arthropods. Here we review how these approaches have already uncovered lineage-restricted regulators, resolved cell-type trajectories, and mapped *cis*-regulatory landscapes across diverse clades. We then take a critical view of their scope and limitations: success depends on high-quality genomes and annotations, careful staging and replication, mitigation of dissociation and ambient-RNA artifacts, and robust cross-species mapping of orthology and cell-type homology. At the regulatory level, linking distal accessible sites to target genes remains a central challenge that often requires integrating chromatin and conformation data with functional perturbations. Progress in this field is further supported by the development and adaptation of enabling tools, such as low-input chemistries (e.g., CUT&Tag), single-nucleus and spatial workflows, and the availability of improved genome assemblies and computational frameworks for multi-omic integration. Ultimately, we argue that the integration of these techniques-especially perturbation with multi-omic data across diverse species-is the key to transforming descriptive regulatory ‘maps’ into a mechanistic understanding of evolution.

## Classical genetic screens and the candidate gene paradigm

The field of arthropod evolutionary developmental biology was founded on the pioneering genetic work carried out in *Drosophila melanogaster* during the 1970s and 1980s (Lewis, 1978; Nüsslein-Volhard et al., 1980; Anderson et al., 1985). Early research addressed two fundamental questions of body plan formation: the identity of individual segments and the mechanism for their periodic generation (Lewis, 1978; Nüsslein-Volhard *et al*., 1980). In 1978, genetic dissection of the Bithorax complex established that clustered homeotic (Hox) loci control segment identity and emphasized the importance of *cis*-regulatory control within a physically linked gene complex (Lewis, 1978). Building on this genetic framework, Nüsslein-Volhard and Wieschaus introduced systematic, genome-wide ethyl methanesulfonate (EMS) mutagenesis screens for embryonic-lethal mutants, identifying 15 loci whose zygotic functions disrupt segment number and polarity-an early example of an unbiased genetic inventory of a developmental program (Nüsslein-Volhard *et al*., 1980).

In the same unbiased screening tradition (including maternal-effect genetics), axis specification emerged as a second major organizing system. (Anderson et al., 1985; Roth et al., 1989). For dorsoventral patterning, Anderson and colleagues showed that the maternal Toll gene product induces dorsoventral polarity, with loss- and gain-of-function alleles producing dorsalized and ventralized embryos, respectively (Anderson *et al*., 1985). Siegfried Roth and colleagues subsequently demonstrated that DV pattern depends on a Toll-controlled gradient of nuclear localization of Dorsal, an NF-κB family transcription factor, generated by maternal “dorsal group” genes and *cactus*; this gradient functions as a morphogen to specify distinct ventral-to-dorsal fates (Roth *et al*., 1989). The same maternal-effect genetic logic also revealed key maternal axis determinants outside DV signaling, including *bicoid* as an anterior morphogen system and *gurken* as a TGFα-like signal required, in the germline, for dorsoventral polarity during oogenesis (Driever and Nüsslein-Volhard, 1988; Neuman-Silberberg and Schüpbach, 1993). Together, these genome-wide screens cemented *Drosophila melanogaster* as the reference framework for comparative developmental genetics and motivated the candidate gene approach in arthropod Evo-Devo: cloning and functional testing of *Drosophila* orthologs in emerging models to assess conservation and evolutionary rewiring of developmental pathways and GRNs.

## Candidate gene approach: Conservation and evolutionary flexibility in axis specification among arthropods

At the turn of the century, candidate-gene studies extended the logic of *Drosophila* genetics across arthropod phylogeny (Patel, 1994; Schröder, 2003). The central question was deceptively simple: if homologous genes underlie homologous structures, how far does the *Drosophila* gene regulatory logic extend-and where does it fail, rewire, or become replaced? This question is not merely comparative; it cuts to the core of how developmental GRNs evolve (Auman and Chipman, 2017).

Crucially, *Drosophila* represents an extreme developmental strategy. As a long-germ insect, most segments are specified nearly simultaneously at the blastoderm stage (Patel, 1994). In contrast, most insects-including the beetle *Tribolium castaneum*-pattern only the anterior early and generate posterior segments sequentially from a segment-addition zone after gastrulation (Lewis, 1978; Patel, 1994; Chipman et al., 2004). In this context, *Tribolium* provides a powerful conceptual bridge: segmentation can be observed as a dynamic, growth-coupled process rather than as a largely pre-patterned event. Candidate-gene studies in this system therefore probe not only conservation, but also the plasticity of regulatory logic under distinct developmental regimes (El-Sherif et al., 2012).

This perspective broadens further when chelicerates are considered. Spider embryos do not simply represent “insects with extra segments.” Their early development proceeds in a cellular environment through a radially symmetric germ disc that breaks symmetry via a migratory signaling center-the cumulus (Akiyama-Oda and Oda, 2003). This embryonic geometry differs fundamentally from the syncytial blastoderm of flies, imposing distinct spatial and temporal constraints on how axis-level signals are deployed and interpreted during development (Akiyama-Oda and Oda, 2006). In this context, candidate-gene comparisons gain explanatory power, as they provide a controlled framework to discriminate deeply conserved molecular components from lineage-specific regulatory architectures.

On the segmentation cascade, early comparative work revealed striking conservation of pair-rule-like gene deployment beyond insects. In the spider *Cupiennius salei*, canonical insect pair-rule genes such as *hairy*, *even-skipped*, and *runt* are expressed in reiterated transverse stripes during posterior elongation, suggesting that key elements of pair-rule logic predate the chelicerate-mandibulate split (Damen et al., 2000). Yet similar phenotypic outputs emerge from distinct regulatory dynamics. In the geophilomorph centipede *Strigamia maritima*, segmentation involves oscillatory or wave-like expression of genes such as *odd-skipped-related* and *caudal*, rather than by fixed genetic stripes laid out in advance as in insects, showing that similar segmented bodies can arise through very different developmental processes (Chipman et al., 2004). These comparisons reveal a central Evo-Devo insight: conservation resides in network components and outputs, not necessarily in wiring or dynamics (Brena and Akam, 2013).

Functional analyses in *Tribolium* reinforced this conclusion. Although canonical pair-rule genes are expressed in striped domains, the regulatory hierarchy established in *Drosophila* does not operate in the same way in the beetle. Similar segmentation patterns are therefore produced by a different regulatory organization, indicating that conservation at the level of gene expression does not imply conservation of the underlying regulatory logic and function. Instead, *Tribolium* segmentation relies on a clock-like, self-regulatory circuit involving *even-skipped*, *runt*, and *odd-skipped*, operating in a sequential manner (Sarrazin et al., 2012; El-Sherif et al., 2012). This shifts the conceptual focus away from rigid classifications such as “primary” versus “secondary” pair-rule genes and toward network dynamics adapted to progressive axis elongation. Here, the candidate-gene approach achieved its greatest strength: it identified conserved actors while simultaneously forcing a re-interpretation of their system-level roles.

The same pattern-conserved components, evolvable wiring-is even more apparent in anteroposterior (AP) axis specification. The canonical example is *bicoid*, a maternal morphogen essential for anterior patterning in flies but absent from most insects (Lemke et al., 2008; Liu et al., 2018). In *Tribolium*, maternally supplied *orthodenticle* (*otd*) and *hunchback* (*hb*) generate strong anterior phenotypes, supporting the hypothesis that an ancestral *otd/hb* system preceded the fly-specific *bicoid* innovation (Schröder, 2003). Importantly, this hypothesis was mechanistically grounded: subtle changes in homeodomain residues, such as the K50 position, can dramatically alter DNA-binding specificity, enabling functional convergence despite sequence divergence (Hanes and Brent, 1989; Liu *et al*., 2018).

However, subsequent analyses complicate a simple “*otd* equals beetle *bicoid*” narrative. In *Tribolium*, much of the apparent early AP function of *Tc-otd* reflects unexpected roles in dorsoventral patterning and cell survival, rendering its morphogen-like role at the blastoderm stage at least partially controversial (Kotkamp et al., 2010). This ambiguity is not a failure of the candidate-gene approach; rather, it exposes its intrinsic limitation. Homologous genes can be co-opted into additional roles, obscuring one-to-one functional analogies. The wasp *Nasonia vitripennis*, where maternal *otd* plays a more direct role in AP patterning, further underscores that different insects can deploy distinct solutions drawn from a shared molecular toolkit (Lynch et al., 2006).

This conflict between conservation and flexibility extends to signaling pathways that polarize the AP axis. In most metazoans, canonical Wnt/β-catenin signaling promotes posterior identity and must be repressed anteriorly to allow anterior structures to form (Lagutin et al., 2003). In *Tribolium*, maternal transcripts of the Wnt negative regulator Axin are localized to the future anterior of freshly laid eggs; knock down of *Tc-axin* via RNAi leads to a graded loss of anterior structures and posteriorization phenotypes. The anterior expansion of *caudal* expression domains at the expense of anterior fates is consistent with ectopic Wnt activation in anterior regions (Fu et al., 2012). These findings demonstrates that the antagonism of Wnt signaling at the anterior pole is required for proper anterior development in a short-germ arthropod, similar in logic to vertebrate anterior head formation where secreted Wnt antagonists repress Wnt activity anteriorly. In *Tribolium*, this repression appears to be mediated intracellularly through Axin’s regulation of β-catenin destruction rather than via secreted antagonists, illustrating yet another way conserved signaling logic can be embedded in a lineage-specific molecular implementation (Fu *et al*., 2012).

**Figure 1 -:**
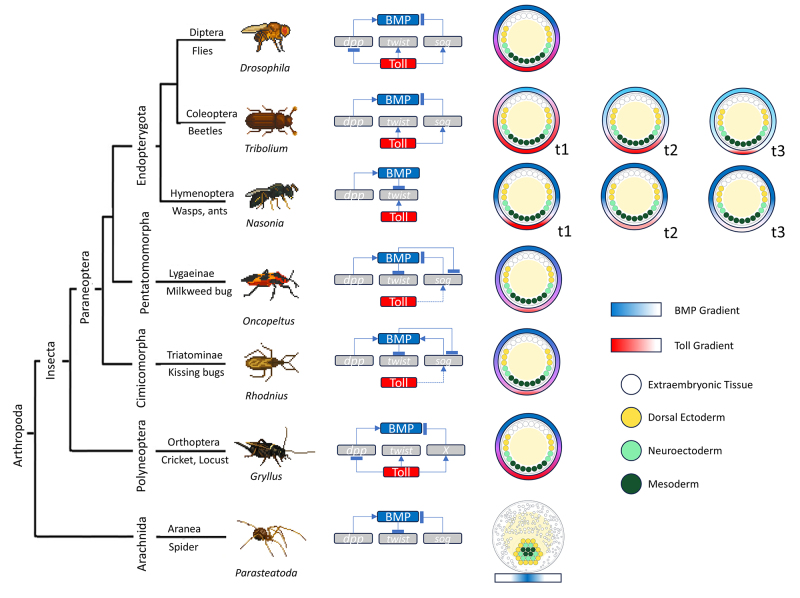
Comparative dorsoventral (DV) patterning architectures across Arthropoda. Left: Simplified phylogeny with one exemplary species per clade. Center: Minimal wiring diagrams summarizing the relative contributions of Toll and BMP pathways to DV patterning in each taxon. Arrows denote positive regulation; “T-bars” denote inhibition. Key components are indicated (Dpp/BMP2/4, Sog/Chordin, Twist as a mesodermal readout, and Toll/Dorsal). Right (t1-t3): Schematic transverse sections (dorsal up) showing the dynamics of the extracellular gradients-BMP (blue) and Toll/Dorsal (red)-and the consequent germ-layer outcomes: extraembryonic tissue (lavender), dorsal ectoderm (yellow), neuroectoderm (light green), and mesoderm (dark green). t1 represents early blastoderm/polarization, t2 peak gradient refinement, and t3 germ-layer allocation. Color intensity indicates relative signaling level. Species rows (top to bottom): Diptera - *Drosophila*: Maternal Toll forms a stable ventral-to-dorsal Dorsal gradient that specifies ventral fates; BMP is secondarily focused dorsally via Sog-mediated shuttling to pattern dorsal ectoderm. Coleoptera - *Tribolium*: Two interdependent, self-organizing circuits-an initially sharp but dissipating Dorsal gradient and a Sog-modulated Dpp gradient-are both required, restoring BMP to a dominant, ancestral role in axis formation. Hymenoptera - *Nasonia*: DV patterning is driven predominantly by a self-organizing BMP gradient with little to no requirement for Toll, illustrating a Toll-independent solution. Hemiptera (Lygaeinae) - *Oncopeltus*: Toll functions chiefly as a symmetry-breaking cue that polarizes a dynamic BMP network, which then executes most DV patterning. Hemiptera (Triatominae) - *Rhodnius*: Maternal Toll is necessary for DV germ-layer specification and interacts with BMP/Sog; BMP holds broad control over DV fates, with Sog modulating BMP distribution. Orthoptera - *Gryllus*: Toll patterns the ventral half and polarizes BMP activity despite the absence of a sog ortholog, revealing a third wiring solution that still yields the canonical Dorsal-high/BMP-high polarity. Araneae - *Parasteatoda*: BMP2/4 (dpp) converts radial to axial symmetry and short gastrulation specifies ventral tissue, a topology analogous-but not identical-to insects.

Dorsoventral (DV) axis specification provides one of the clearest demonstrations that evolutionary conservation in development operates primarily at the level of signaling modules, not fixed regulatory hierarchies (Figure 1). While the molecular players involved in DV patterning-most notably Toll/NF-κB and BMP signaling-are broadly conserved across arthropods, their relative weights, temporal deployment, and system-level logic vary strikingly among lineages (Pechmann et al., 2021; Roth, 2023).

In *Drosophila*, DV patterning is dominated by a maternal Toll signaling cascade that establishes a stable nuclear gradient of the transcription factor Dorsal (Roth et al., 1989). This gradient directly specifies ventral and lateral cell fates at the syncytial blastoderm stage, while BMP signaling (via *decapentaplegic, dpp*) is largely restricted to the dorsal domain and refines dorsal ectodermal fates. In this system, Toll functions as the primary patterning pathway, and BMP acts downstream as a secondary player. Outside flies, however, this hierarchy is not conserved.

In spiders, DV axis formation occurs within a fundamentally different embryonic geometry: a radially symmetric germ disc that breaks symmetry through the migration of the cumulus, a BMP-expressing signaling center (Akiyama-Oda and Oda, 2003). Here, BMP signaling is not merely a dorsal refinement cue but is essential for the radial-to-axial transition itself, while *short gastrulation (sog)* specifies ventral fates (Akiyama-Oda and Oda, 2006). BMP-*sog* antagonism is conserved in spiders, but its deployment occurs in a spatial and temporal regime distinct from the fly syncytial blastoderm. This reflects a general Evo-Devo pattern: conserved signaling logic operates across divergent morphogenetic contexts.

Findings from *Tribolium castaneum* further highlight how DV patterning can be reorganized around self-regulatory network modules. In contrast to *Drosophila*, the nuclear Dorsal gradient in *Tribolium* is transient, shrinking rapidly and disappearing during early development. Rather than acting as a stable morphogen, Toll signaling initiates DV polarity, which is then maintained and refined by a robust BMP/Dpp network modulated by extracellular regulators such as *sog*, *twisted gastrulation (tsg)* and *tolloid (Tld)*. Functional perturbations reveal that BMP signaling plays a far more central and indispensable role in DV patterning in *Tribolium* than in flies, consistent with a system that relies on dynamic feedback and self-organization rather than a fixed maternal gradient (Chen et al., 2000; Chipman et al., 2004; Fonseca et al., 2008; Nunes da Fonseca et al., 2010).

Additional insect lineages reveal yet further rewiring of the same conserved modules. In the cricket *Gryllus bimaculatus*, Toll signaling, as in *Drosophila,* has a direct role in ventral patterning and in polarizing BMP activity, but strikingly does so in the absence of an identifiable *sog/chordin* ortholog (Pechmann et al., 2021). This demonstrates that the classical BMP-Sog antagonistic module, while common, is not strictly required; alternative molecular solutions can achieve equivalent patterning outputs.

In hemimetabolous insects such as *Oncopeltus fasciatus*, Toll signaling primarily serves to polarize a dynamic BMP network rather than acting as the dominant patterning signal (Sachs et al., 2015). In the wasp *Nasonia vitripennis*, DV polarity relies almost entirely on BMP signaling, with little to no contribution from Toll, representing an extreme shift in pathway “division of labor” (Buchta et al., 2013). Finally, in the hemipteran *Rhodnius prolixus*, Toll signaling is once more required for DV patterning but also contributes to anterior-posterior embryo positioning, while *sog* exhibits lineage-specific functional diversification, including a pro-BMP activity (Berni et al., 2014, 2023). In *Rhodnius*, even the canonical role of a conserved antagonist is evolutionarily labile.

Taken together, DV patterning across arthropods reveals a striking principle: conservation resides in the availability of signaling modules, not in their hierarchical deployment. Toll and BMP pathways can function as primary patterning signals, secondary refiners, polarity cues, or self-organizing feedback systems, depending on embryological context and evolutionary history. This flexibility allows developmental systems to accommodate changes in egg architecture, cleavage mode, and morphogenetic movements without abandoning deeply conserved molecular components (Pechmann et al., 2021).

From an Evo-Devo perspective, DV axis specification thus mirrors the lessons from AP patterning and segmentation. Candidate-gene approaches successfully identify conserved actors, but they repeatedly demonstrate that network topology, timing, and feedback structure are free to evolve. Understanding how DV patterning systems change, therefore, requires moving beyond gene presence/absence toward comparative analyses of network dynamics, signal integration, and self-regulation transition that naturally motivates the integration of candidate-guided functional studies with unbiased genomic and systems-level approaches.

Together, these AP and DV case studies converge on a central conclusion. Candidate-gene approaches are powerful because they start from mechanistic hypotheses grounded in evolutionary and biochemical insights, revealing which nodes of a developmental GRN are constrained. At the same time, they repeatedly show that conserved components can be reweighted, recombined, or supplemented by lineage-specific innovations. As a result, candidate gene logic alone cannot reconstruct how developmental systems evolve.

To understand evolutionary dynamics of arthropod GRNs-and how developmental novelty emerges-candidate-guided functional work must be integrated with unbiased discovery approaches, including comparative genomics, transcriptomics, and systems-level network inference across broad taxonomic sampling. In this sense, candidate gene studies defined the ceiling of a reductionist view, setting the stage for the next generation of Evo-Devo research that bridges gene-centric hypotheses with network- and system-level logic.

## The new toolkit: Unbiased omics for non-model arthropods

The ‘omics’ era enabled unbiased approaches such as whole-genome sequencing, transcriptomics, and functional screens. These unbiased approaches revealed novel developmental regulators beyond the established candidate-gene list (see below).

### Bulk RNA-seq unlocks hidden toolkits

Bulk RNA-seq has proven transformative because it couples an unbiased, genome-wide view of transcription with simple, inexpensive sample preparation (Mortazavi et al., 2008; Marioni et al., 2008; Sultan et al., 2012), a stark contrast to the gene-by-gene approaches of the past. As soon as reference genomes outside Diptera became available, for example, the 200 Mb *Tribolium castaneum* assembly in 2008 (*Tribolium* Genome Sequencing Consortium, 2008) and the compact 176 Mb *Strigamia maritima* genome in 2014 (Chipman et al., 2014), researchers could finally map stage-specific read sets in non-model embryos with single-exon precision, bringing the full developmental time-course of these animals into focus.

In the short-germ beetle *Tribolium*, densely sampled RNA-seq series spanning the syncytial blastoderm to late germ-band stages recovered >12 000 expressed genes and revealed hundreds of transcripts from the posterior segment addition zone (SAZ) (Khan et al., 2019). RNA-seq revealed transient peaks in *maelstrom* expression during SAZ formation, hinting at unexplored roles beyond its canonical function in the piRNA pathway, and dozens of previously unannotated zinc-finger and homeobox factors with no clear *Drosophila* orthologs. This expanded the canonical segmentation GRN far beyond the familiar ‘pair-rule’ genes (Pridöhl et al., 2017). Time-resolved clustering of the same dataset uncovered waves of oscillatory expression of *even-skipped* and *odd-skipped* that phase-shift across successive samples, independently confirming a vertebrate-like segmentation clock operating in both blastoderm and germ-band contexts of the beetle embryo (El-Sherif et al., 2012). 

In a recent study, Reding et al. (2024) exemplify the strength of unbiased strategies in Evo-Devo (Reding *et al*., 2024). Instead of restricting their analysis to known orthologs of *Drosophila* pair-rule genes, the authors combined transcriptomic profiling with a large-scale *in situ* hybridization screen to identify regulators of segmentation in the milkweed bug *Oncopeltus fasciatus*. This approach led to the unexpected identification of *Blimp1* as a pair-rule gene-even though *Blimp1* plays no such role in *Drosophila melanogaster*. Using both RNAi and CRISPR-Cas9 mutagenesis, the authors demonstrated that *Blimp1* is required for alternate segment specification, revealing that while the logic of pair-rule patterning is evolutionarily conserved, the specific genes implementing this logic can differ markedly across insect lineages. More broadly, this study demonstrates that genome-wide, assumption-free approaches can uncover hidden regulatory components, challenge conclusions derived from classical model organisms, and clarify patterns of developmental system drift under evolutionary constraint (True and Haag, 2001; Reding *et al*., 2024). 

A parallel effort in the geophilomorph centipede *Strigamia* married its new genome to staged mRNA profiles and high-resolution *in-situ* hybridization screens. The resulting time-course showed that a conserved core of primary pair-rule genes (*eve, runt, odd, hairy*) is switched on in a double-segment rhythm even before gastrulation (Chipman et al., 2004; Brena and Akam, 2013), but that at least 40 centipede-specific transcription factors and signalling components join the network as segmentation proceeds-many of them encoded in gene families lost from insects altogether (Brena and Akam, 2013; Chipman *et al*., 2014). 

In a seminal study, Yoon et al. (2019) investigated axis formation by bulk transcriptome of sectioned moth, flies and mosquitoes’ eggs and found that, unlike *Drosophila*, which uses *bicoid*, these insects rely on ancient, conserved genes such as *odd-paired*, *cucoid*, and *pangolin* (Yoon *et al*., 2019). Localized maternal transcript isoforms of these genes establish anterior polarity (Yoon *et al*., 2019). This polarity control is achieved not by changes in protein sequence but through alternative transcription generating isoforms with distinct untranslated regions that enable anterior targeting (Yoon *et al*., 2019). The study highlights how conserved developmental outcomes can emerge from different molecular mechanisms, illustrating developmental systems drift and emphasizing the importance of transcript regulation as a driver of Evo-Devo innovation (True and Haag, 2001; Yoon *et al*., 2019). Altogether, these studies highlight how bulk RNA-seq can uncover lineage-restricted regulators that would be invisible to candidate-gene search anchored in *Drosophila*.

**Figure 2 -:**
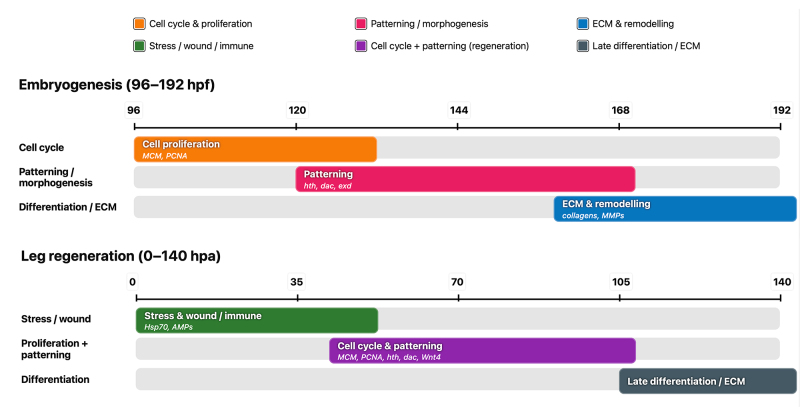
Temporal modules of *Parhyale* leg embryogenesis versus regeneration. Schematic comparison of dominant transcriptional/functional modules during embryonic leg development (top; 96-192 hpf, hours post-fertilization) and post-amputation leg regeneration (bottom; 0-140 hpa, hours post-amputation). Colored bars denote the approximate onset and duration of module activity inferred from marker genes (labels inside bars); tick marks indicate time. Embryogenesis: An early cell-cycle/proliferation phase (orange; ~96-140 hpf; markers: MCM complex, PCNA) is followed by patterning/morphogenesis (magenta; ~132-170 hpf; markers: hth, dac, exd), culminating in ECM deposition and tissue remodeling (blue; ~160-192 hpf; markers: collagens, MMPs) as differentiation proceeds (grey background track). Regeneration: An injury-triggered stress/wound/immune module (green; ~0-35 hpa; markers: Hsp70, antimicrobial peptides) precedes a combined cell-cycle + patterning phase (purple; ~35-105 hpa; markers: MCM, PCNA, hth, dac, Wnt4). Regrowth ends with late differentiation/ECM (dark grey; ~105-140 hpa). Color key (top left): orange-cell cycle & proliferation; magenta-patterning/morphogenesis; green-stress/wound/immune; purple-cell cycle + patterning (regeneration); blue-ECM & remodeling; dark grey-late differentiation/ECM. Abbreviations: ECM, extracellular matrix; MMPs, matrix metalloproteinases; MCM, minichromosome maintenance; PCNA, proliferating cell nuclear antigen; hth, dac, exd, limb patterning genes. Bars are qualitative and not to scale.

Lastly, bulk transcriptomics has also illuminated how developmental toolkits are redeployed in new life-history contexts. Comparative RNA-seq of regenerating versus developing legs in the crustacean *Parhyale hawaiensis* showed that although ~80 % of “regeneration” transcripts are reused from embryogenesis, the order of deployment is radically rewired: early patterning genes such as *hth* and *dac* are delayed, whereas stress and ECM modules activate sooner, underscoring a regeneration-specific temporal logic (Sinigaglia et al., 2022). Follow-up single-nucleus RNA-seq resolved these shifts at cell-type resolution and revealed fibroblast and immune cell populations unique to regenerating legs. Meanwhile, an embryo-wide microRNA atlas suggested that post-transcriptional control helps retune the common genetic toolkit for use in repair (Calvo et al., 2022; Almazán et al., 2022) (Figure 2).

In the honeybee *Apis mellifera*, analyses of bulk RNA-seq together with small-RNA profiles during the cleavage stages revealed the presence of pri-miRNAs and a TAGteam-like *cis*-regulatory motif already at the onset of development. These findings indicate an unusually early activation of the zygotic genome and a markedly accelerated maternal-to-zygotic transition, suggesting that haplodiploidy is associated with a shifted temporal logic of genome activation rather than a simple reuse of canonical insect MZT programs (Pires et al., 2016).

Taken together, these studies help explain why bulk RNA-seq so rapidly became the central experimental workhorse of Evo-Devo beyond *Drosophila*. With relatively modest sequencing effort, bulk transcriptomics provides an unbiased window into developmental gene expression, allowing the identification of previously unrecognized regulatory players, precise inference of the timing of developmental programs, and the formulation of testable functional hypotheses. In practice, this strategy can convert a newly sequenced arthropod into a tractable functional system within only a few well-chosen developmental stages, effectively collapsing the gap between genome availability and mechanistic developmental insight.

### Single-cell atlases resolve cellular heterogeneity

Single-cell transcriptomics is reshaping arthropod Evo-Devo by replacing tissue-level averages with explicit maps of gene expression across cell types, developmental time, and differentiation trajectories (Trapnell et al., 2014; Luecken and Theis, 2019). Rather than treating “the embryo” as a homogeneous entity inferred from bulk profiles, scRNA-seq and snRNA-seq describe development as a structured population of heterogeneous cellular states. This framework enables the detection of rare lineages, transient progenitors, and branching trajectories that are largely inaccessible to pooled measurements. (Luecken and Theis, 2019; Saelens et al., 2019). 

This shift has been driven by scalable platforms that routinely profile thousands to tens of thousands of cells per sample, either through droplet-based microfluidics (e.g., Drop-seq and 10x-style barcoding) or higher-sensitivity full-length protocols such as Smart-seq2. These approaches build on the earliest demonstrations of single-cell whole-transcriptome sequencing (Tang et al., 2009; Picelli et al., 2013; Macosko et al., 2015; Zheng et al., 2015). For arthropods in particular, single-nucleus methods provide a practical solution to dissociation challenges in lipid-rich or mechanically resistant tissues. At the same time, nuclei-based profiling systematically under-represents cytoplasmic transcripts and therefore can differ from whole-cell scRNA-seq in detectable ways (Habib et al., 2017; Bakken et al., 2018; Denisenko et al., 2020; Ding et al., 2020). 

The common house spider *Parasteatoda tepidariorum* provides a clear example of how cell atlases expand comparative Evo-Devo beyond *Drosophila*-centric candidate genes. Early single-cell and single-nucleus studies of very early embryos showed that genome-wide expression patterns are sufficient to reconstruct embryo-scale polarity, offering an unbiased entry point into axis formation without presupposing a restricted marker set (Akiyama-Oda et al., 2022). Subsequent snRNA-seq datasets spanning late stage 5 to stage 7 captured the onset of segmental patterning along the anterior-posterior axis, resolving segmentation-related transcriptional states at single-cell resolution in a non-insect arthropod (Akaiwa et al., 2025).

Later-stage atlases further demonstrated how such datasets inform both cell-type diversity and gene-family evolution. In stages 7-9, a scRNA-seq atlas identified 20 transcriptionally distinct clusters and recovered many known developmental genes alongside numerous previously uncharacterized loci (Leite et al., 2024). Hox genes frequently marked specific clusters, and Hox ohnologs, generated by the ancestral arachnopulmonate genome duplication, were often segregated into different clusters, consistent with sub- and/or neo-functionalization detectable at the level of cell states. (Schwager et al., 2017; Leite *et al*., 2024). Extending the developmental series to stages 10-12 increased the number of resolved clusters to 24 and incorporated cell types associated with organogenesis, including neural subtypes, cardiac-related cells, and morphogenetic structures such as the ventral sulcus (Medina-Jiménez et al., 2024). Together, these datasets illustrate that single-cell atlases do not simply add resolution; they recast questions such as segmentation, head patterning, and limb development in terms of cell-state transitions and lineage-specific regulatory modules rather than fixed sets of canonical markers (Leite *et al*., 2024; Medina-Jiménez *et al*., 2024; Akaiwa et al., 2025).

Single-cell approaches are equally informative for regeneration, where similar anatomical outcomes may arise through distinct developmental dynamics. In the amphipod crustacean *Parhyale hawaiensis*, leg regeneration was long hypothesized to reuse embryonic patterning genes with altered timing. A study integrating microanatomy with snRNA-seq demonstrated that regenerated legs restore complex structure and sensory function, and that regenerated and uninjured legs are essentially indistinguishable in their cell-type composition and transcriptional profiles (Almazán et al., 2022). This conclusion depends on demonstrating cell-type completeness rather than gene-level similarity and would be difficult to support using bulk RNA-seq alone (Almazán *et al*., 2022).

In hemimetabolous insects, where segmentation proceeds sequentially and segment addition zone dynamics differ from the long-germ strategy of *Drosophila*, comprehensive single-cell atlases are still limited. Nevertheless, quantitative datasets combining morphometrics, cell-division patterns, and gene expression already illustrate the same conceptual advantage. In *Oncopeltus fasciatus*, coupling segmentation morphology with proliferation profiles and marker expression revealed the posterior segment addition zone as a structured and dynamic field, rather than a uniform posterior domain defined by a small candidate-gene set (Auman et al., 2017; Auman and Chipman, 2018). Such datasets provide an important bridge to full single-cell atlases by anchoring transcriptional states to measurable cellular behaviors (Auman *et al*., 2017). 

High-resolution atlases in dipterans and disease vectors further show how single-cell resources connect Evo-Devo to physiology and host-pathogen interactions. In the mosquito *Aedes aegypti*, a scRNA-seq atlas of the adult female midgut resolved major cell types involved in digestion, immunity, and vector competence, providing a cellular framework for blood-meal biology (Wang et al., 2024). Similarly, a large atlas of the *Drosophila* larval central nervous system profiled over 100,000 cells across multiple stages and resolved diverse neuronal and glial populations, offering a reference for nervous system maturation in a genetically tractable model (Corrales et al., 2022). As non-model arthropod atlases accumulate, they increasingly enable direct cell type-to-cell type comparisons across deep evolutionary distances, an approach that was largely inaccessible under candidate-gene frameworks (Luecken and Theis, 2019; Saelens et al., 2019).

Finally, several limitations must be considered. Cell dissociation or nuclei isolation disrupts spatial organization and immediate cell-cell interactions that are often central to developmental mechanisms, including morphogen gradients, juxtacrine signaling, and mechanical constraints (Luecken and Theis, 2019; Denisenko et al., 2020). Most atlases also represent discrete timepoints; while pseudo time inference can be informative, it relies on assumptions and cannot replace direct lineage tracing or live imaging (Trapnell et al., 2014; Saelens et al., 2019). Technical issues such as dropout, ambient RNA, doublets, stress responses, and systematic differences between nuclei- and cell-based measurements can further bias inferred trajectories if not carefully controlled (Denisenko *et al*., 2020; Ding et al., 2020; Young and Behjati, 2020).

### Chromatin and *cis* regulation enter the arena

The shift from measuring which genes are transcribed to asking how those transcriptional decisions are made has been driven by chromatin-level assays that operate without predefined candidate genes or loci. Methods such as Omni-ATAC-seq (Buenrostro et al., 2015; Corces et al., 2017; Merrill et al., 2022), ChIP-seq (Visel et al., 2009) and their low-input derivatives now make it possible to track enhancer accessibility, histone modifications, and higher-order chromosomal organization across embryogenesis. Together, these approaches provide access to regulatory layers that were largely inaccessible during the era of gene-by-gene cloning.

The comprehension of how *Cis*-Regulatory Elements (CRE), genomic sequences can activate weak promoters, in particular enhancers, has moved beyond the confines of classical *Drosophila* genetics. In *Tribolium castaneum*, *cis*-regulatory discovery pipelines combining locus selection with reporter assays established one of the first systematic workflows for enhancer analysis in a non-model insect (Lai et al., 2018; Tomoyasu and Halfon, 2020). When paired with chromatin-level methods such as ATAC-seq and ChIP-seq, these approaches enable enhancer discovery even in species where stable transgenesis remains difficult. Parallel work in flies has further shown that enhancer architecture encodes dynamic transcriptional outputs, generating propagating or oscillatory expression patterns rather than static domains (Bothma et al., 2014; Keller et al., 2020) Together, these developments bridge classical *cis*-regulatory assays with genome-wide chromatin profiling and provide a general framework for linking enhancer activity to developmental patterning across arthropods.

In the amphipod *Parhyale hawaiensis,* Omni-ATAC-seq across ten developmental stages identified more than 60,000 accessible regions. Many of these lie near crustacean-specific limb and gill genes and become accessible precisely at the onset of appendage outgrowth, directly linking *cis*-regulatory activation to morphological innovation without the need for transgenic reporter lines (Corrales et al., 2022). 

Spiders provide a clear example of how chromatin profiling can be integrated with functional genetics to probe early regulatory control. In *Parasteatoda tepidariorum*, ATAC-seq performed on embryos subjected to parental RNAi against the early-acting factor *fuchi*, a rapidly evolving GATA factor, revealed thousands of genomic regions that fail to become accessible upon knockdown (Iwasaki-Yokozawa et al., 2022). This widespread failure of chromatin opening is a hallmark of pioneer transcription factor activity, factors that initiate zygotic genome activation (ZGA) by binding compacted chromatin and enabling subsequent regulatory input. In insects such as *Drosophila* and *Tribolium*, this pioneer role is fulfilled by Zelda (Liang et al., 2008; Ribeiro et al., 2017), whereas vertebrates rely on factors such as Pou5f3, SoxB1, and Nanog (Miao et al., 2022). In spiders, the data therefore suggest that *fuchi* acts as a lineage-specific pioneer or pioneer-like regulator of ZGA. More broadly, this supports a model in which conserved developmental transitions are maintained through different upstream regulators in different lineages, illustrating developmental systems drift at the level of genome activation. Integration of these ATAC-seq data with stage-matched single-cell RNA-seq further allowed accessible elements to be assigned to defined cell types, yielding one of the first cell-type-resolved enhancer atlases outside insects (Leite et al., 2024).

Histone-based profiling has advanced in parallel. ChIP-seq targeting the centromere-specific histone variant CENH3 in *Tribolium castaneum* uncovered meta polycentric centromeres spanning approximately 40% of each chromosome, an organization that could not be inferred from cytological data alone and that challenges prevailing assumptions about chromosome segregation in Coleoptera (Gržan et al., 2020).

Because conventional ChIP-seq requires large amounts of material and high-quality antibodies, many studies now rely on CUT&RUN or CUT&Tag, assays that use protein A-Tn5 fusions and only a few thousand cells (Hainer and Fazzio, 2019; Kaya-Okur et al., 2019). These approaches have enabled multi-omic analyses in non-model arthropods, including a recent study of the Colorado potato beetle that combined CUT&Tag, RNA-seq, and enzymatic methyl-seq to profile H3K36me3, H3K27ac, and CpG DNA methylation (Länger et al., 2025).

In most animals, CpG DNA methylation is established by DNMT3, the *de novo* DNA methyltransferase that writes new methylation marks onto previously unmethylated DNA during development, while DNMT1 primarily maintains these marks during replication. The Colorado potato beetle lineage has secondarily lost DNMT3, meaning that new CpG methylation cannot be written in the canonical way. This loss makes the species a natural experiment for testing how DNA methylation is patterned and interpreted in the absence of standard *de novo* methylation machinery (Länger et al., 2025). Despite the absence of DNMT3, the study found a strong genome-wide correlation between H3K36me3 and gene-body CpG methylation. This relationship mirrors patterns described in vertebrates, where H3K36me3 guides DNA methylation to actively transcribed gene bodies, and is consistent with comparative evidence that *Drosophila* orthologs of methylated invertebrate genes are enriched for H3K36me3 (Dhayalan et al., 2010; Nanty et al., 2011; Baubec et al., 2015). Together, these results indicate that the coupling between histone modifications and DNA methylation can be evolutionarily rewired, persisting or re-emerging even after the loss of DNMT3. Whether this coupling reflects a conserved regulatory function or a by-product of transcriptional activity in insects remains an open question

Three-dimensional genome organization adds a critical regulatory dimension to Evo-Devo by revealing how genes and their regulatory elements are physically arranged and interact within the nucleus during development. Hi-C measures genome-wide chromatin contact frequencies, allowing the reconstruction of higher-order features such as chromosome territories, topologically associating domains (TADs), and long-range enhancer-promoter interactions. Unlike transcriptomic approaches, which describe what genes are expressed, Hi-C defines which regulatory interactions are physically possible by constraining enhancer-promoter communication in three-dimensional space. In *Parasteatoda tepidariorum*, Hi-C scaffolding upgraded the genome assembly to twelve chromosome-level pseudomolecules and uncovered domain architectures that bracket Hox clusters and neurogenic loci, indicating that long-range chromatin insulation-rather than local enhancer evolution alone-contributes to spider body-plan diversification (Zhu et al., 2023). For Evo-Devo, the key contribution of Hi-C is that it links regulatory sequence evolution to three-dimensional genome architecture. By defining which enhancers can physically contact which promoters, Hi-C constrains the space of regulatory interactions and helps explain how conserved gene sets can be deployed differently across lineages. Because Hi-C operates at kilobase-scale resolution with moderate sequencing effort, it can be integrated with ATAC-seq and CUT&Tag to follow regulatory elements from chromatin opening, through histone modification, to target-gene contact within a unified framework (Deng et al., 2022). Together, chromatin-level assays shift Evo-Devo from identifying conserved genes to mapping regulatory space. By resolving when and where enhancers become accessible, histone marks are deposited, and chromosomes fold, these approaches define the substrates on which mutation and selection act. This makes it possible to link sequence-level change to morphological diversification across arthropods without reliance on stable transgenic lines. As technical constraints related to tissue input and antibody availability continue to diminish, comparative “regulome” analyses across spiders, centipedes, and beetles are likely to become routine.

### Spatial transcriptomics restores anatomy

Spatial transcriptomics (ST) refers to a class of methods that measure gene expression while preserving the spatial location of transcripts within intact tissues. Unlike bulk or single-cell RNA-seq, which require dissociation and therefore lose anatomical context, ST retains information about where genes are expressed within an organ or embryo, allowing transcriptional programs to be interpreted directly in relation to morphology. In sequencing-based ST, thin tissue sections are placed on barcoded oligo-dT capture arrays, imaged after histological staining (often H&E), and processed *in situ* for reverse transcription before library preparation and sequencing. Because each sequencing read carries a spatial barcode, transcript counts can be projected back onto the tissue image, generating genome-wide expression maps that preserve tissue architecture. Conceptually, this extends classical *in situ* hybridization from one gene at a time to transcriptome-scale measurement in a single experiment (Ståhl et al., 2016).

Spatial resolution has advanced rapidly, and it is useful to distinguish physical feature size from the binning strategies often applied to increase sensitivity. Standard 10x Genomics Visium uses 55 μm barcoded capture spots, whereas Visium HD replaces discrete spots with a contiguous grid of 2 × 2 μm barcoded squares that are typically aggregated computationally (Habern, 2019). Slide-seq and Slide-seqV2 introduced bead-based arrays with ~10 μm resolution, approaching cellular scale and improving capture efficiency in the V2 chemistry (Rodriques et al., 2019; Stickels et al., 2021). Stereo-seq, based on DNA nanoball-patterned arrays, achieves submicron feature spacing and can support single-cell-scale atlases when binned appropriately for tissue type and sequencing depth (Chen et al., 2022). Increasingly, benchmarking efforts enable evidence-based comparisons across platforms, protocols, and sample types, including fresh-frozen and Formalin-Fixed, Paraffin-Embedded (FFPE) workflows. 

In arthropods, ST has moved beyond methodological refinement to generate biological insights that are difficult to obtain from scRNA-seq or bulk RNA-seq alone. First, ST anchors transcriptional states back onto anatomy, allowing scRNA-seq clusters to be interpreted in spatial terms. In adult *Drosophila melanogaster*, a targeted high-plex spatial approach mapped 150 transcripts across body sections and localized multiple cell-type signatures *in situ*. Notably, spatial mapping revealed unexpected subcellular mRNA patterning within the large flight muscle cells, information that is typically lost during tissue dissociation (Janssens et al., 2025).

Second, ST directly links transcriptional specialization to tissue microanatomy and functional output. In the orb-weaving spider *Larinioides sclopetarius*, an integrated analysis combining scRNA-seq, spatial transcriptomics, histology, and proteomics showed that the silk gland secretory epithelium comprises six cell types arranged into three spatial zones. These zones produce distinct combinations of silk proteins whose secretions remain segregated and correspond to the layered structure of the final fiber, providing a direct connection between spatial gene regulation and biomaterial architecture (Sonavane et al., 2024). 

Technical constraints remain, particularly for arthropods: small tissue size, cuticle-associated pigmentation and autofluorescence, and RNA preservation in yolk-rich embryos. However, these challenges are increasingly manageable as protocols diversify, FFPE-compatible workflows mature, and community benchmarks clarify best practices. From an Evo-Devo perspective, the key advance is that gene regulatory network logic can now be projected onto the physical body plan at scale, accelerating hypothesis generation in emerging model systems where spatial context is essential.

## Conclusions, perspectives and current limitations

Unbiased genomics, transcriptomics, and epigenomics have reshaped arthropod Evo-Devo by making comparative developmental biology feasible beyond a narrow set of laboratory model systems (*Tribolium* Genome Sequencing Consortium, 2008; Chipman et al., 2014; Schwager et al., 2017; Panfilio et al., 2019). The central shift is not simply increased data volume, but the ability to connect cell identity, regulatory DNA, and gene-expression dynamics across development, regeneration, and environmental response. This integration enables explicit hypotheses about how gene regulatory networks (GRNs) are wired and how they change over evolutionary time (Macosko et al., 2015; Zheng et al., 2017; Ma *et al*., 2020). In practice, these approaches loosen the historical *Drosophila*-centric bottleneck by allowing spiders, myriapods, crustaceans, and hemimetabolous insects to be interrogated at comparable molecular resolution, provided that reference genomes, annotations, and sampling designs are adequate (Lewis, 1978; Nüsslein-Volhard et al.,1980).

Crucially, each experimental advance has been accompanied by the development of dedicated computational analytical frameworks. For chromatin accessibility, ATAC-seq (Buenrostro et al., 2015) provides a rapid route to candidate CREs, but comparative inference depends on robust peak calling, normalization, and motif or footprinting strategies that tolerate variable read depth and genome quality. Similarly, the transition from conventional ChIP-seq to low-input alternatives such as CUT&RUN (Hainer and Fazzio 2019) and CUT&Tag (Kaya-Okur et al., 2019) was not merely incremental: these methods enabled chromatin profiling in small samples and even at single-cell resolution, while introducing method-specific biases such as antibody dependence and accessibility-driven background-that require explicit controls. On the single-cell side, transcriptomic atlases are now routine, but their explanatory power depends on computational tools that move beyond cluster catalogues toward regulatory mechanism, including integration, batch correction, trajectory inference, and GRN reconstruction (Trapnell et al., 2014; Corrales et al., 2022).

As a result, the field increasingly relies on multi-modal integration and formal regulatory inference. Joint profiling methods that measure chromatin accessibility and gene expression in the same cell-such as sci-CAR (Cao et al., 2018) and SHARE-seq (Ma et al., 2020)-directly link CRE candidates to putative target genes. Complementary algorithms such as Cicero (Pliner et al., 2018) formalize peak-peak and peak-gene associations through co-accessibility. In parallel, single-cell GRN inference approaches, including SCENIC (Aibar et al., 2017), identify transcription factor regulons associated with cell-type or cell-state transitions. More recent frameworks explicitly incorporate chromatin information to constrain and test regulatory models; for example, CellOracle integrates scRNA-seq and scATAC-seq to infer cluster-specific GRNs and simulate transcription factor perturbations in silico (Kamimoto et al., 2023). Together, these approaches provide a route from correlated gene expression to mechanistic, testable network hypotheses.

Despite these advances, key limitations remain and must be stated clearly because they bound current inference. First, uneven phylogenetic sampling and sparse taxonomic coverage bias comparative claims, and incomplete genomes or annotations complicate cross-species CRE and orthology analyses. Second, peak-to-gene assignment remains probabilistic: co-accessibility improves prioritization but does not substitute for functional validation, particularly for long-range or context-dependent enhancers. Third, single-cell assays introduce systematic distortions: dissociation or nuclei isolation can under-represent fragile cell types, ambient RNA and doublets confound rare populations, and scATAC-seq sparsity limits motif and footprint inference. Fourth, chromatin profiling remains constrained by antibody specificity and epitope accessibility; CUT&RUN and CUT&Tag reduce background and input requirements but do not eliminate antibody-driven bias. Finally, spatial transcriptomics (Ståhl et al., 2016) and three-dimensional genome mapping (Hi-C; Lieberman-Aiden et al., 2009) add anatomical and topological context, but resolution, capture efficiency, and computational integration across modalities remain limiting.

A realistic path forward therefore goes beyond compiling candidate enhancers or correlated gene modules and can be articulated as a sequence of tractable steps. (1) Build anchored references: generate developmental time series and cell-type atlases using scRNA-seq and multiome approaches, linked to curated orthology and improved genome assemblies. (2) Map regulatory logic: integrate chromatin accessibility, transcription factor binding proxies (CUT&RUN/CUT&Tag), and expression to infer candidate regulatory edges (TF → CRE → target gene), while explicitly quantifying uncertainty. (3) Introduce causality: prioritize perturbation-based validation, using RNAi or CRISPR *in vivo* where possible, and pooled perturbation-single-cell assays where systems permit (e.g., Perturb-seq; Dixit et al., 2016). (4) Compare networks across species: treat GRNs as evolvable entities by comparing topology, enhancer turnover, and modularity across lineages and developmental contexts, rather than relying solely on gene-level conservation. Together, this pipeline converts descriptive datasets into a mechanism-focused comparative framework.

Finally, expanding taxonomic breadth is not only an intellectual goal but a methodological necessity. Evolutionary novelties-alternative segmentation modes, axis-patterning strategies, and regenerative capacities-often arise in lineages that remain poorly sampled. Biodiversity-rich regions, including Brazil, provide an opportunity to reduce phylogenetic bias and connect Evo-Devo to conservation and One Health questions, provided that sampling, permitting, and capacity building are conducted ethically and sustainably. Coupled with multi-modal inference and perturbation-based validation, this expansion positions arthropod Evo-Devo to explain not only how GRNs generate form, but why regulatory architectures diverge across the arthropod tree.

## Data Availability

 This is a review article and no new datasets were generated or analyzed during the preparation of this manuscript. All information discussed in the article is based on previously published literature, which is cited in the reference list.
